# Spiritual, religious, and existential concerns of children and young people with life-limiting and life-threatening conditions: A qualitative interview study

**DOI:** 10.1177/02692163231165101

**Published:** 2023-03-28

**Authors:** Hannah May Scott, Lucy Coombes, Debbie Braybrook, Anna Roach, Daney Harðardóttir, Katherine Bristowe, Clare Ellis-Smith, Julia Downing, Fliss EM Murtagh, Bobbie Farsides, Lorna K Fraser, Myra Bluebond-Langner, Richard Harding

**Affiliations:** 1 Florence Nightingale Faculty of Nursing Midwifery and Palliative Care, Cicely Saunders Institute, King’s College London, London, UK; 2 Royal Marsden NHS Foundation Trust, London, UK; 3 Louis Dundas Centre for Children’s Palliative Care, University College London, London, UK; 4 International Children’s Palliative Care Network, Kampala, Uganda; 5 Wolfson Palliative Care Research Centre, Hull York Medical School, University of Hull, Hull, UK; 6 Brighton and Sussex Medical School, University of Sussex, Brighton, UK; 7 Martin House Research Centre, Department of Health Sciences, University of York, York, UK; 8 Rutgers University, New Brunswick, NJ, USA

**Keywords:** Child, palliative care, spiritual concerns, existential concerns, religious concerns, terminal illness

## Abstract

**Background:**

Despite being a core domain of palliative care, primary data on spiritual and existential concerns has rarely been collected among children with life-limiting and life-threatening conditions and their families. Existing evidence has tended to focus on the religious aspects among children with cancer.

**Aim:**

To identify the spiritual needs of children with life-limiting and life-threatening conditions.

**Design:**

Cross-sectional semi-structured, qualitative interview study with children, families and health and social care professionals. Verbatim transcripts were analysed using Framework analysis

**Setting/participants:**

Purposively sampled children with life-limiting and life-threatening conditions, their parents and siblings, health and social care professionals recruited from six hospitals and three children’s hospices in the UK, and commissioners of paediatric palliative care services recruited through networks and a national charity.

**Results:**

One hundred six participants were interviewed: 26 children (5–17 years), 53 family members (parents/carers of children 0–17 years and siblings (5–17 years)), 27 professionals (health and social care professionals and commissioners of paediatric palliative care). Themes included: living life to the fullest, meaning of life and leaving a legacy, uncertainty about the future, determination to survive, accepting or fighting the future and role of religion. Children as young as 5 years old identified needs or concerns in the spiritual domain of care.

**Conclusions:**

Addressing spiritual concerns is essential to providing child- and family-centred palliative care. Eliciting spiritual concerns may enable health and social care professionals to identify the things that can support and enhance a meaningful life and legacy for children and their families.

**What is already known about the topic?**

- Although spiritual concerns are recognised as a core component of palliative care for children, there is a paucity of primary data.
- Self-report data from children is rare, and existing evidence is largely proxy data from parents or health and social care professionals and mainly focused on the religious aspect of spiritual care for cancer patients.

**What this paper adds?**

- Specific spiritual concerns among children with a range of life-limiting and life-threatening conditions and their families (parents and siblings) included: living life to the fullest, meaning of life and leaving a legacy, uncertainty about the future, determination to survive, accepting or fighting the future and role of religion.
- This work broadens understanding of the spiritual domain for these children beyond religious needs to existential and value-based spiritual concerns.
- Recognition of the way in which children conceptualise spirituality and being able to identify their spiritual concerns is essential for child- and family-centred holistic palliative and end-of-life care.

**Implications for practice, theory or policy:**

- Professionals can optimise children and family’s wellbeing through identification of the things that provide meaning for them, and working together to set goals and actions towards achieving them.
- Such concerns must be assessed beyond religious considerations.
- Simple tools and training to support professional may be useful in implementing this.

## Background

Children and young people living with life-limiting and life-threatening conditions have many complex symptoms and concerns that span health and social care domains.^1^ Paediatric palliative care is a holistic approach that aims to address and manage the symptoms and concerns of children and young people with life-limiting and life-threatening conditions and their families.^2^ The goal is to identify and improve symptoms and concerns across four core domains: physical, psychological, social and spiritual.^3^

Whilst there has been considerable debate as to what constitutes spirituality in adult palliative care, one of the more accepted definitions is that: ‘Spirituality is the aspect of humanity that refers to the way individuals seek and express meaning and purpose and the way they experience their connectedness to the moment, to self, to others, to nature, and to the significant or sacred’.^4^ Moreover consensus work by the European Association of Palliative Care (EAPC) Reference Group on Spiritual Care states that spiritual care requires consideration of people’s values (e.g. what is most important for each person), faith and beliefs, and existential concerns (e.g. concerns surrounding meaning of life, hope and death).^5^ However spirituality in children and thus the extent to which this definition meets the needs of children with life-limiting and life-threatening conditions is less well understood.^6^

Spirituality for dying children is often linked to the losses they face relating to their sense of normality or that which is normal for them.^6^ Thus spiritual care should be about supporting children and families with meaning-making and redefining hope, whether that is in a religious sense or not.^6^ Spiritual care for children and young people has also been defined as that which addresses and resolves spiritual or existential distress such as fear or questions like ‘why me?’, as well as supporting them to find meaning and exploring legacy.^7^ This paper accepts the definition of spirituality as put forward and agreed upon for adult palliative care^4,8,9^ but extends it to also recognise the importance of hope and normality in spirituality for children.^6,7^

In a recent systematic review on spiritual care in palliative care, only two of 53 included studies were of paediatric populations and neither study included primary data from children.^10^ Similarly, a systematic review of symptoms and concerns of children with life-limiting and life-threatening conditions found that of the 37/81 studies that included spiritual concerns, nearly all focused on cancer and recruited professionals or parents as proxies. Moreover, much of the existing literature has focused more on religious aspects of spirituality.^11,12^ As part of a larger study to develop and test outcome measurement for children and young people facing life-limiting illness^13^ this article aims to identify and describe the components of the spiritual domain of palliative care for children with life-limiting and life-threatening conditions as expressed by the children themselves, their families, professionals and commissioners.

## Methods

### Research question

What are the core spiritual, existential and religious concerns of children, young people and their families facing life-threatening or terminal illness?

### Design

A semi-structured, qualitative interview study was conducted from a critical realist perspective^14^ and reported in accordance with the consolidated criteria for reporting qualitative studies (COREQ).^15^ This data was collected as part a study aiming to identify priority outcomes for care for children with life-limiting and life-threatening conditions and their families^13^ as part of the larger Children’s Palliative care Outcome Scale (C-POS) study aimed at developing and validating a child- and family-centred outcome measure for implementation into routine paediatric palliative care.

### Population

Inclusion criteria: Children (5–17 years) with any life-limiting condition; parents/carers with a child <18 years old with a life-limiting condition; siblings (5–17 years) of children with a life-limiting condition; healthcare professionals with >6 months experience of caring for children with life-limiting conditions; commissioners of UK paediatric palliative care services.

Exclusion criteria: children unable to communicate via an in-depth interview or by using ‘draw and talk’ or play methods or via their parents, and/or speak a language not supported by NHS translation services, and/or currently enrolled in another study, and/or unable to give consent/assent; parents/carers and siblings unable to give consent/assent, and/or speak a language not supported by NHS translation services.

### Setting

Six hospitals and three children’s hospices in the UK across England and Northern Ireland.

### Sampling

Children and their families were purposively sampled to ensure representation of a wide range of ages and conditions as evidence suggests that the symptoms and concerns that matter to children and young people vary by age and diagnosis.^1^

### Recruitment

Children and their families were identified by their care teams at the nine recruiting sites during their weekly multidisciplinary team meetings, during ward rounds or during outpatient appointments. Eligible families were then approached by a member of their care team who would introduce the study to them and request them to consider participation. If they expressed an interest to learn more about the study, child age-specific information sheets and the parent/caregiver information sheets were provided. Families had 2 weeks to decide whether to proceed and clarify if they would like to contact the researcher direct (email, telephone), or whether they would like the researcher from King’s College London to make contact.

Healthcare professionals were identified by the site service manager who approached staff with information on the study and took verbal consent to share their contact details with research team members. Commissioners of UK paediatric palliative care services were recruited via the study’s partner professionals and a national children’s palliative care non-governmental organisation.

### Data collection

Semi-structured interviews were conducted by LC (experienced children’s palliative care nurse, new to qualitative research), AR (experienced in working with children, new to qualitative research) and DB (experienced qualitative researcher) between April 2019 and September 2020. All interviewers received training and supervision on conducting interviews with children, including the legal, ethical and communication issues.

Interviews commenced with demographic questions and children were asked about their hobbies and interests to build rapport. ‘Draw and talk’ and play methods were used, with toys, paper and pencils provided to children to aid interviews where required to facilitate the interviews. A topic guide with open questions informed by a systematic review of symptoms and concerns in children with life-limiting conditions^1^ and the World Health Organisation (WHO) definition of paediatric palliative care^16^ was used to ensure all domains of the WHO definition of palliative care was discussed.

Interviews were audio-recorded, transcribed verbatim and pseudonymised.

### Data analysis

Full transcripts were deductively and inductively coded^17,18^ by LC, DB, AR, HS (experienced qualitative researcher, experienced in working with children), and DH (experienced in working with children, new to qualitative research) using NVivo 12 Software.

Analysis followed the five-step Framework method^17,18^; familiarisation, coding, developing an analytical framework, applying the framework, charting and interpretation. Interviews were analysed individually, and the within stakeholder groups, across age ranges, and then across stakeholder groups. The use of Framework analysis enabled comparison and contrast of findings across the sample both within and across stakeholder groups. As part of robust and valid outcome measurement development, recommendations^19^ and guidelines^20^ emphasise the importance of key stakeholder views to ensure face and content validity. It is only by looking across different stakeholder groups that similarities and differences between groups can be identified. Importantly, some groups may overlook something that they do not consider important or may find difficult to talk about. Thus, whilst different participants both within and across groups may view or understand the construct of spirituality in different ways, it is important to understand it from all of their perspectives to support the delivery of person-centred care for children that addresses spiritual concerns.

Regular meetings were held to discuss emerging themes and resolve any differences (20% of transcripts were independently coded by two researchers). RH, KB and CES were consulted and resolved if disagreement in coding and interpretation arose. Analysis was reviewed by the study steering group throughout the study.

### Ethical issues

Ethical approval was granted by the Bloomsbury research ethics committee (HRA:19/LO/0033). Participants over 16 years old provided written informed consent. Those with parental responsibility provided written informed consent for participants<16 years. Those <16 years provided written assent.

## Results

### Sample characteristics

One hundred and four interviews were conducted with *N* = 106 participants (two parents and two siblings were interviewed together): *n* = 26 children, *n* = 53 family members, *n* = 15 health and social care professionals and *n* = 12 commissioners (see Table 1). To protect anonymity of children with rare conditions, International Classification of Diseases-10 (ICD-10) chapter headings are reported in lieu of precise diagnoses.^13^

**Table 1.** Participant demographic characteristic.

	*N* or mean (range)
Children (*n* = 26)	
Age (years)	12 (5–17)
Gender	
Female:Male	17:9
Diagnosis	
Gastrointestinal	10
Cancer	6
Neurological	5
Congenital	3
Metabolic	1
Respiratory	1
Family members (*n* = 53)	
Age (years)	32 (5–65)
Gender	3
Female:Male	7:16
Relationship to child	
Mother	29
Father	10
Sister	7
Brother	6
Sibling Caregiver	1
Diagnosis of child	
Neurological	17
Congenital	10
Metabolic	10
Cancer	6
Gastrointestinal	6
Infectious disease	2
Genitourinary	1
Perinatal	1
Age of child with life-limiting condition (years)	10 (0–17)
Professionals (*n* = 27)	
Gender	
Female:Male	25:2
Profession	
Commissioner of paediatric palliative care	12
Nurse	7
Doctor	3
Chaplain	1
Physiotherapist	1
Play specialist	1
Psychologist	1
Social worker	1

## Main findings

**Figure 1.**
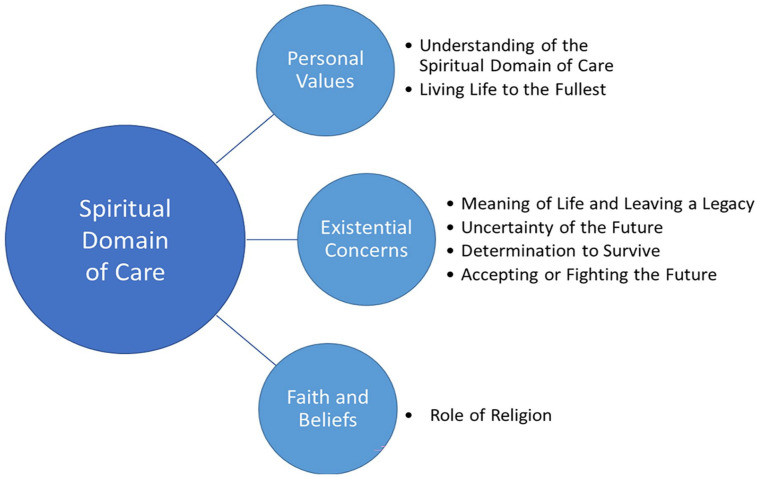
Spiritual domain of care.

The framework analysis identified seven themes that relate to the three dimensions of the spiritual domain of care as defined by the EAPC Reference Group on Spiritual Care^5^: (1) Relating to the term ‘spiritual’, (2) living life to the fullest, (3) meaning of life and leaving a legacy, (4) uncertainty of the future, (5) determination to survive, (6) accepting or fighting the future, and (7) role of religion. Figure 1 shows how these themes relate to the three dimensions of the spiritual domain of care as defined by the EAPC reference group.^5^

## Dimension 1: Personal values

### Theme 1: Relating to the term ‘spiritual’

Some health and social care professionals discussed a lack of confidence in asking about spiritual concerns, and noted that in terms of asking children and families about spiritual needs, this often focused only on religious needs. This was also reflected in parents understanding of spiritual needs with parents often responding to questions about spiritual needs in terms of religion

I think sometimes when we say spiritual needs I think er we look more at the religious side but that does not always reflect spiritual needs *[Oncology nurse]*

Erm yeah [parent] and I are not remotely sort of religious. So erm, no we don’t really have any. I mean, spiritual sort of needs *[Mother of child aged 8 with congenital condition]*

When asked directly about spiritual concerns, parents stated that they and their children did not have needs in this domain

Interviewer: Do you think that (child) has any spiritual concerns or any like concept of spirituality?

No, I don’t think he has any. . .any concept of that whatsoever, not that I’ve. . .no I don’t think, no. . .no *[Father of child aged 12 with congenital condition]*

### Theme 2: Living life to the fullest

Being able to take part in activities they enjoyed was very important to children. Being able to do the things that made their life meaningful, and retained some degree of normality, contributed to ‘living life to the fullest’.

Well I’m happy because I get better, but then I’m sad because I miss school, miss my friends, miss my family *[Child aged 12 with Respiratory condition]*

Interviewer: what do you mean by living your life

Doing what I want in my life *[Child aged 9 with gastrointestinal condition]*

Health and social care professionals and commissioners recognised this theme as important, and wanted to help enable children to live their lives to the fullest through goal setting and supporting them to continue to do the things that were important to them.

you know that they’re en. . .enabled to live the. . .the fullest life that they’re able to do. Do you know what I mean that they. . .they can achieve some of their goals and development and. . .and as appropriate to them *[Commissioner]*

for their quality of life it is about going to school and being around friends and trying to be normal *[Psychologist]*

For parents this was often talked about in terms of ensuring that their children had quality of life, particularly in terms of comfort and happiness.

We can’t change his length of life, but I can do everything I can to make sure his quality of life is good and that he lives his best life possible *[Mother of child aged 2.5 with metabolic condition]*

## Dimension 2: Existential concerns

### Theme 3: Meaning of life and leaving a legacy

Parents and children often had questions such as ‘why me?’. Professionals and parents also highlighted that children expressed concern that they had done something wrong to deserve their illness

You know ‘why me?’ and we had a lot of anger first off, again the issue I just said ‘Oh you know “eat your veg, fruit and veg, you know you’ll be big and strong” you know’, ‘drink lots of water because it’s good for you’ erm. . .and initially we had the “well you lied to me, why. . .you know why, why me. Why, what have I done wrong?”’ *[Father of 13-year-old child with a gastrointestinal condition]*

I think one of the things that children will often worry about. . .about things that they feel they’ve done wrong [. . .] those sort of things really do play very heavily on erm. . .small children and particularly if they’d had some kind of religious background maybe from, you know perhaps going to a faith school or. . .or having RE lessons at school. *[Hospital Chaplain]*

Children also wanted their life to mean something and to leave a legacy after they had died. This included setting up charity initiatives, inspiring other children and leaving messages for their family members

I don’t think there’s much point in going through life and just supporting yourself, because when you go through. . .at the end of life, you’re gonna think, what have I done for other people? Because those are the people, you’re gonna miss and you’re think, what have I actually done for them. If you haven’t done anything for them, you may regret it [. . .] I can write a story, I can include that and hopefully help other people to make that kind of change *[Child aged 17 with gastrointestinal condition]*

I did quite a lot of work about her bucket list and wanting to and her legacy and wanting to, she wanted to leave messages for her brother and other people in her family *[Social worker and family therapist]*

### Theme 4: Uncertainty about the future

Uncertainty about the future was a concern for both children and their families.

There was longer-term uncertainty around their prognosis, how long they had to live, and worry about dying, particularly for older children. Parents and one sibling also expressed similar concerns

I felt worried when I was, like really sick in [month]. Umm, yeah, I was just worried like [long pause] and then [pause] like, was I gonna get through it and like stuff like that. Because, that time was really [pause] wasn’t the best *[Child aged 15 with gastrointestinal condition]*

And I think particularly the older kids, although there’s sort of an understanding that their life, well they’re 15 or they’re 16, they understand what’s going on, they are often the most frightened because they have complete understanding. *[Transplant Nurse]*

They don’t know how long he has *[Mother of child aged 1 with congenital condition]*

Interviewer: Okay and what would you say worries you and what’s your biggest worry about. . .?

Umm . . .that erm. . .she might die *[Sibling of Child aged 14 with Neurological condition]*

However, the unpredictable nature of life-limiting and life-threatening conditions often created uncertainty in the more immediate short-term. This made it difficult for children and their families to make plans due to unpredictable changes in symptoms

You can’t really arrange things because you might arrange something for this week or the next week and then you get to that week and you have got an infection and you are in hospital or something like that. So it changes. . . so you can’t really plan much. *[Child aged 15 with cancer]*

we literally take every day as it comes. We don’t plan anything. We just do day by day which is all you have to do with a sick child *[Mother of child aged 4 with congenital condition]*

### Theme 5: Determination to survive

Children voiced a determination to live their lives and survive despite their health conditions, wanting to be brave and overcome their prognosis

It’s ok because I am brave *[Child aged 5 with Gastrointestinal condition]*

with cancer you want to live, you want to show people that you can overcome this *[Child aged 13 with cancer]*

the fact that I’m still here means that my journey hasn’t finished *[Child aged 17 with metabolic condition]*

Parents also spoke of their children’s determination in terms of being brave or fighting, despite their condition and prognosis

she’s classed as an end of life stage now, but she’s plodding on, she’s still got her fight *[Mother of child aged 8 with neurological condition]*

I think part of it is that she feels like she doesn’t want people to know she’s not coping or she, you know, she doesn’t want people to know that she’s been upset or you know. There’s still that stigma behind it, about being brave *[Mother of child aged 12 with cancer]*

He has got such a strong heart. He keeps fighting back. He is a fighter. And you know, you have to give him a lot of credit for that. He’s a strong little soul *[Mother of 10 year old with neurological condition]*

### Theme 6: Accepting or fighting the future

Older children were often more aware of the life-limiting nature of their condition and prognosis. Whilst some were accepting others expressed internal conflict over accepting the possibility or actuality of death whilst simultaneously wanting to fight to keep going and survive

you just keep going until you reach that final choice or decision of what’s going to happen to you, either you can overcome this or a different consequence that everyone hopes will never happen *[Child aged 13 with Cancer]*

If they realise that they are, that they are going to die, you know, they feel distraught cos they are not going to have a life that others have. They are more aware. The young ones are not so aware of what they are going to miss out on, what they are not going to achieve, whereas the older ones, they know. They can see, they know what they’re going to lose *[Oncology nurse]*

if you talk about (child) dying he’s just like ‘I don’t wanna talk about it because it’s not gonna happen yet’ do you know what I mean *[Mother of 14 year old with metabolic condition]*

Parents also discussed difficulty in accepting the life-limiting aspect of their child’s diagnosis, and how challenging it was to come to terms with knowing their child would die

the biggie is obviously the. . .the life-limiting diagnosis and the fact that we’re gonna lose him *[Mother of 7 year old with metabolic condition]*

Irrespective of their condition and prognosis, children still wanted to think about and make plans both for the short-term and the longer-term future

I wanna be grown up *[Child aged 15 with neurological condition]*

I would wanna travel like around the world *[Child aged 15 with gastrointestinal condition]*

The teenager that died recently, I mean she was still going to do her GCSE’s this summer. And she died much quicker than we thought. But no, she was definitely going to still do them. *[Nurse]*

## Dimension 3: Faith and religion

### Theme 7: Role of religion

A number of parents and health and social care professionals discussed children’s religious beliefs and needs. Whilst some professionals and parents spoke of children wanting to continue to be able to attend church, many parents spoke in terms of passive identification of their own or their children’s beliefs rather than in terms of unmet religious needs

I had one boy that was very concerned about. . .he wanted to spend time with umm. . .his priest. . .erm and I found that. . .that was. . .that was unusual for me because he was quite young, so that really sort of jarred for me. I’ve had old. . .I’ve had adolescents who have really wanted to make sure they could still attend. . .erm church and things like that *[Oncology Clinical Nurse Specialist]*

I think she’d put herself down as Buddhist but, obviously not practicing *[Father of child aged 16 with cancer]*

Only one child participant spoke about their religious faith and beliefs. They considered their religion a source of strength and comfort and it was important for them to be able to continue to attend church

I’m a Christian, so I believe in Jesus and that he’s my lord and saviour [. . .] as I’ve gone through all of these. . .all of this and I’ve been in hospital. . .erm I always remember that, you know there as someone who suffered even worse for me [. . .]there’s a greater hope and like the greater hope is in Jesus and that I trust in that. You know even whatever happens, whether you know I die or whether I live, it’s for ‘Him’ and you know I’m just gonna continue to live a life according to his grace *[Child aged 17 with gastrointestinal condition]*

Similarly for some parents, religion could be a source of comfort and their faith became stronger, even influencing their willingness to discuss their child’s care. However other parents and health and social care professionals spoke of parents having a crisis of faith or losing their faith altogether due to their child’s ill health. In cases when one parent held onto their faith and the other lost it, religion could become a source of conflict

the families don’t want to discuss anything because their faith is so strong. They are going to be saved *[Children’s Community Nurse]*

I do believe in God and you know sort of and erm the guys here do know I have a faith even though I have thought you know long and hard at times. And had sort of inward arguments with you know with whoever it is that’s up there. But I have to hold on to something *[Mother of child aged 10 with neurological condition]*

I mean there’s been recent erm. . .situation where there’s been a Greek. . .Greek Orthodox family and they were. . .erm going through a period of great spiritual distress and also confusion. . .erm because within the family, one of the people was Greek Ortho. . .Orthodox, the other one wasn’t. . .erm in just the period of the child’s illness, one parent had become more religious, the other one had become less religious *[Hospital Chaplain]*

For some families, religion could also be a source of anger (particularly when they did not have religious belief). This was often linked to existential concerns and questions relating to the meaning or reason for developing a life-limiting or life-threatening condition

(child) finds it really frustrating when people say ‘oh I’ll pray for you’. . . because actually it’s [. . .] ‘what God would do this’. . . so when people start saying ‘We’ll pray for you’ and things like that, we think. . .. And often we both just ignore it, we don’t comment on it but I know (child) does find it a bit frustrating *[Father of child aged 16 with cancer]*

## Discussion

### Main findings

This novel primary data provided by children, their family members and professionals has given detailed understanding of a previously under-explored domain. When asked directly about spiritual concerns parent/carers tended to state that they and/or their children did not have spiritual concerns, or responded to the question in terms of religion. However, when probed further, particularly in relation to worries, hopes or other things that were important to them, children and their families discussed many concerns that demonstrate the experience of the dimensions of spirituality in line with the definition of spirituality for children discussed at the beginning of this article.^6,8^

Professionals tended to lack confidence in asking about spiritual concerns, particularly existential concerns and tended to focus more on the religious aspects of spirituality. However, the data revealed that even if asked using broad terms of ‘spirituality’, families may interpret this to mean religion. The data demonstrate that children and families do have spiritual concerns to be addressed (although notably, only one sibling discussed spiritual concerns in the interviews). These concerns may need to be asked about in a less direct way; for example, asking them about the things that are important to them, their hopes, and worries, rather than if they have any spiritual or religious needs or concerns.

### What this study adds

Whilst some of the themes echoed those identified in a recent systematic review,^1^ such as meaning of life and determination to survive, other novel, important themes were identified. It was particularly important for children to live life to the fullest through being enabled and supported to continue to engage in activities that were important to them and that gave their lives meaning. These included taking part in their hobbies, going to school and seeing friends and family. These highlight the importance of continuing normality and maintaining meaning as core components of spiritual wellbeing.^13^

Understanding the way in which children and families conceptualise and express spiritual concerns is essential to assessment and planning. Additional training for professionals may also be beneficial to develop their confidence and support them in asking about spiritual concerns. As is a strength of this work in including the voices of children in an area where their perspectives are often lacking, it is recommend that the perspectives of children be included in training and development for professionals to help overcome the pervasive view that children do not have spiritual needs. Fear of causing distress, or not having a solution, should never be a reason for clinicians not asking about spiritual concerns. Instead of directly asking if children and their families have spiritual or existential concerns, professionals should consider shaping discussions about spiritual and existential needs in the context of what matters to the child, and what they value in terms of their usual routines, in order to talk about their life, future and spiritual or existential concerns and build confidence in talking about and addressing these concerns. Moreover, professionals need to be aware of the potential for faith to become a source of conflict between family members in order to be able to best support families.

### Strengths and weaknesses

This research explored a previously under-researched area despite the well documented ethical challenges in conducting primary research with this population relating to difficulties in gaining ethics approval and clinician gatekeeping.^21,22^ Previous studies in this area have tended to rely on proxy reports from parents/carers and professionals, or focused solely on children with a cancer diagnosis.^1^ A major strength of this study is the involvement of children themselves from the age of 5 years old, and with a range of life-limiting conditions. The sample size was also relatively large in comparison to other studies that include children with life-limiting conditions, and the geographical spread of participant recruitment across the UK. However there were also limitations. There were only a small number of UK recruitment sites and one site only recruited children with gastrointestinal diagnoses, reflected in the larger number of participants from this group. Moreover a lack of ability to extensively probe in interviews with children within a larger study to identify the breadth of their symptoms and concerns means that some concerns may have been missed or underexplored. Whilst interviews were adapted for children’s age and developmental stage, additional child-centred techniques (such as using photographs or puppets) may have been able to better elicit children’s concerns and needs.^23–25^

## Conclusion

If care is to be child and family-centred, then spiritual concerns should be identified and addressed equally to other domains of care. Children have spiritual concerns when facing life-limiting and life-threatening conditions, and, when explored appropriately, have the capacity to articulate these. For professionals in practice, it is important work with children and their families to identify the things that are most important to them and that give their lives meaning, and to work together to set achievable goals that can support a meaningful life and legacy.
